# p53 Normal and p53 Abnormal Clear Cell Ovarian Carcinomas: Clinical Characteristics and Biomarker Profiles

**DOI:** 10.1097/PGP.0000000000001191

**Published:** 2026-05-27

**Authors:** Piret Hellberg, Annukka Pasanen, Jonna Similä-Maarala, Ralf Bützow, Heini Lassus

**Affiliations:** * Department of Obstetrics and Gynecology, Gynecologic Oncology; † Department of Pathology; ‡ Department of University of Helsinki and Helsinki University Hospital, Helsinki, Finland

**Keywords:** Clear cell ovarian carcinoma, p53 normal, p53 abnormal, Biomarkers, Survival

## Abstract

A new concept of molecular classification for clear cell ovarian carcinoma (CCOC) has been proposed: *TP53* wild-type and *TP53* mutated. Our aim was to evaluate this classification at the immunohistochemical level in p53 normal and abnormal subgroups. Clinicopathologic factors and 12 immunohistochemical biomarkers (p53, PR, ER, β-catenin, vimentin, ARID1A, HNF1-β, E-cadherin, c-erb-B2, MIB-1, p16, and L1CAM) and patient prognosis were analyzed in 132 CCOCs. The p53 abnormal group presented with significantly worse disease-specific overall survival (DSS) and disease-free survival (DFS) than the p53 normal group, which was largely due to higher stage and a more frequent presence of residual tumor in the p53 abnormal group. Furthermore, higher stage and presence of residual tumor were markers of poor outcome within both p53 subgroups. Interestingly, the presence of ascites was related to shorter DSS only in p53 normal cases. The p53 normal group was also characterized by a higher frequency of ARID1A loss and HNF1-β positivity, whereas p16 overexpression and ER positivity were more common in p53 abnormal cases. Interestingly, ARID1A loss correlated with poor DSS in the p53 abnormal group. In the p53 normal tumors, ER positivity was associated with better DSS and p16 positivity with worse DFS. L1CAM positivity was associated with residual tumor only in the p53 normal group. Our findings support the existence of 2 distinct molecular subgroups of CCOC, p53 normal and p53 abnormal, with diverse characteristics and patient outcomes. Clinical and molecular differences were discovered within these subgroups.

Clear cell carcinoma represents a distinct histopathologic subtype of ovarian carcinoma, accounting for ∼10% of all cases, with a substantially higher incidence reported in Asian populations (up to 25%). Endometriosis is a known risk factor for clear cell as well as for endometrioid carcinoma, another equally common subtype. In contrast to high‑grade serous carcinoma, clear cell carcinoma is typically diagnosed at an earlier stage and at a younger age. Although this stage distribution contributes to a more favorable overall prognosis, outcomes in patients with recurrent or advanced disease remain poor, largely due to the inherent chemoresistance characteristic of this subtype.^1^ To better understand the variable behavior of clear cell ovarian carcinoma and develop new treatment options, knowledge of the underlying molecular mechanisms and their clinical correlations is needed.

The most frequent molecular aberrations in clear cell ovarian carcinoma (CCOC) involve mutations in the tumor suppressor gene *ARID1A* (30%–60%) and the oncogene *PIK3CA* (30%–50%), alterations that may act synergistically during carcinogenesis. A large international consortium recently reported targeted deep sequencing of 163 putative CCOC driver genes in 421 primary tumors, complemented by whole-transcriptome sequencing of 211 of these cases. The analysis revealed 2 molecular subclasses of CCOC: the first subclass (83%) was enriched with the typical canonical CCOC genes (eg, *ARID1A* mutations), and the second was largely comprised of *TP53*-mutated tumors (17%)^2^. The first subclass demonstrated upregulation of genes involved in anaerobic metabolic pathways and was associated with early-stage disease and a history of endometriosis. The second group was enriched with genes involved in extracellular matrix organization and mesenchymal differentiation. The tumors in the second group were more likely to be advanced stage and present with poor outcome.^3^ The independent prognostic significance of p53 expression was further confirmed in a large OTTA (ovarian tumor tissue analysis) consortium study including 748 CCOCs, in which abnormal p53 expression was significantly associated with overall survival in both univariate and multivariate analyses.^4^

We previously published a work on endometriosis-associated ovarian carcinomas, testing the TCGA molecular classification used in endometrial carcinoma on these tumor types.^5^ In clear cell carcinoma, the prognosis of the p53 abnormal group (20% of cases) was poor as compared with the NSMP group (76% of cases). In CCOC, POLE, and MMRd subtypes are uncommon and present with excellent outcome, closer to NSMP than p53 abnormal tumors.^2,5^

As the proposed molecular classification of clear cell ovarian carcinoma into “classic CCOC” and “TP53-mutated CCOC” is relatively recent, data comparing clinical characteristics across these subclasses remain limited. The prevalence and prognostic significance of biomarkers within these groups may also differ. Our objective was, therefore, to investigate clinicopathologic features, immunohistochemical biomarkers, and disease outcomes in p53 abnormal versus p53 wild-type clear cell ovarian carcinoma.

## MATERIALS AND METHODS

### Patients

At the outset, the study included 140 patients treated for clear cell ovarian carcinoma at the Department of Obstetrics and Gynecology of Helsinki University Central Hospital between January 1, 1989 and December 31, 2013. This is a tertiary hospital where the treatment of ovarian cancer is centralized. Successive patients treated for clear cell ovarian carcinoma were searched according to the pathologic records. Approval from the Ethics Committee of the Helsinki University Hospital and from the National Supervisory Authority of Welfare and Health was obtained. The clinical information of the patients was collected from the hospital records, and additional survival information was gathered from the Population Register Center. This material has been described in detail in our previous work.^6^

All cases were reviewed by a pathologist specialized in gynecologic pathology (R.B.). The histopathology of the cases was originally re-evaluated based on criteria set by the WHO Classification of Tumours of Female Reproductive Organs (4th Edition 2014).^7,8^ Additional re-evaluation with immunohistochemistry (including WT-1, HNF1-β, Napsin A, p16, and PR) was performed for ambiguous cases.^7,9^ After re-evaluation, 132 clear cell carcinomas remained in the cohort. The tumors were staged according to the 2009 FIGO staging system.^8^ Response to therapy was assessed after the initial 6 to 8 chemotherapy cycles. Patients who did not receive chemotherapy were evaluated 5 to 6 mo after the primary surgery. Disease-specific overall survival (DSS) was calculated from the date of diagnosis (primary surgery) to death from ovarian carcinoma. Disease-free survival (DFS) was estimated for patients who had a complete response following the primary treatment. In all, 33 of the 132 patients had residual disease at completion of treatment, and 98 had complete remission. In 1 patient, follow-up data were unavailable. In all, 53 of the 132 patients included in this study died from ovarian cancer, and 79 patients died from other causes or were alive at the end of the follow-up period. Patients who died of other causes or were alive at follow-up were censored. The median follow-up time for patients who were censored at the end of the follow-up was 9.3 yr (range: 1.7–25.3 yr).

### Tissue Microarray Construction

Histologic slides were analyzed by a gynecologic pathologist and representative areas of each tumor were marked on 1 to 2 slides. Four 0.8 mm cores were drawn from the corresponding area of the paraffin blocks and were inserted in the recipient TMA block using a manual tissue microarrayer (Beecher MTA-1, Beecher Instruments).

### Immunohistochemistry

We used the following monoclonal antibodies for immunohistochemistry (IHC): ERa (SP1, Roche/Ventana), PR (16, Novocastra), p53 (DO-7, Dako), p16 (E6H4, CINtec Histology), MIB-1 (MIB-1, Dako), Her2 (4B5, Roche/Ventana), E-cadherin (HECD-1, Invitrogen), L1CAM (SIG-3911, Covance, clone 14.10), ARID1A (HPA005456, Sigma-Aldrich), beta-catenin (CAT-5H10, Zymed), HNF1b (CLO374, Atlas Antibodies), vimentin (V9, Dako), and WT1 (6F-H2, Cell Marque).

A pathologist blinded to clinical data (A.P. and J.S.-M.) performed the scoring. Problematic cases were examined by a second investigator (R.B.), and a consensus was reached. Samples with ambiguous stainings were discarded. Examples of immunohistochemical stainings are presented as Supplemental Digital Content 1–3, http://links.lww.com/IJGP/A241, http://links.lww.com/IJGP/A242, http://links.lww.com/IJGP/A243.

p53: Abnormal p53 staining (p53 abn) was defined as diffuse and strong nuclear staining (>80% of cells), completely negative (“null”) staining, or cytoplasmic staining in carcinoma cells. Heterogeneous and weak staining was classified as wild-type expression.^9^

PR and ER: The cut-off for PR/ER positivity, that is, >10% of the tumor nuclei staining with any intensity, was adopted based on breast cancer and endometrial carcinoma studies.^10,11^

β-catenin: Membranous staining was considered normal; abnormal staining was defined as diffuse or focal nuclear positivity.

Vimentin: Any quantity of cytoplasmic staining was scored positive.

ARID1A: Staining was considered negative when tumoral cells presented diffuse or clonal type loss of nuclear expression. As indicated by a previous mutational study, a heterogeneous “checkerboard” pattern of staining and diffuse nuclear staining was classified as positive.^12^

HNF1-β: Any quantity of nuclear staining of at least moderate intensity was classified as positive.

E-cadherin: Loss of E-cadherin was defined as clonal or diffuse lack of membranous staining; positive or weakened staining was classified as normal.

HER2/c-erb-B2: Membranous staining was classified as positive or negative. Positive: Tumor presents complete, intense circumferential membranous staining in >10% of tumor cells (IHC 3+, strong positive) or weak to moderate complete membrane staining detected in >10% of invasive tumor cells (IHC 2+). Negative: Incomplete faint membrane staining and within >10% of invasive tumor cells (IHC 1+) or no staining seen or incomplete faint/barely perceptible membrane staining within ≤10% of invasive tumor cells (IHC 0).

MIB-1: The proportion of carcinoma cells presenting nuclear staining of any intensity was scored as for breast cancer: negative (<5%), low (5%–14%), moderate (15%–29%), and strong (≥30%). In dichotomous comparisons, strong positivity was compared with samples with MIB-1 <30%.

p16: Positive result was described as strong and diffuse (>50% of cells) nuclear and cytoplasmic staining (diffuse-block type staining), and entirely negative, focal and mosaic stainings were considered negative.

L1CAM: Expression was scored as defined before,^6^ with ≥10% of membranous staining considered positive.

### Statistical Analysis

We analyzed categorical variables with the Pearson χ^2^ test and the Fisher exact test. The exact number of eligible patients for each analysis is included in the tables and figures. Overall, the number of missing values were small: only 0 to 3 samples per biomarker were not interpretable. The disease-specific overall survival and disease-free survival were calculated using the Kaplan-Meier method, and the log-rank test was used to compare differences between groups. For multivariate survival analysis, the Cox proportional hazards model was used. Statistical significance was set at *P*<0.05. Data were analyzed using IBM SPSS version 29 software. To correct for the multiple hypothesis testing, the false discovery rate (FDR) was tested using the Benjamini-Hochberg method.

## RESULTS

### Distribution of Clinicopathologic Characteristics in p53 Normal Versus p53 Abnormal Groups

Of the 129 interpretable clear cell ovarian carcinomas, 104 (81%) were p53 normal (wild type) and 25 (19%) were p53 abnormal. p53 abnormality was significantly associated with higher disease stage and presence of residual tumor in primary surgery (Table 1).

**TABLE 1:** Clinicopathological Characteristics in p53 Normal and p53 Abnormal Groups

	n (%)			
	Total	p53 Normal	p53 Abnormal	*P* ^*^
No. patients	129	104 (81)	25 (19)	
Age at diagnosis				
<58 yr (median)	64 (50)	53 (51)	11 (44)	0.53
≥58 yr (median)	65 (50)	51 (49)	14 (56)	
Disease stage				
I	76 (59)	72 (69)	4 (16)	**<0.001**
II	4 (3)	3 (3)	1 (4)	
III	33 (26)	22 (21)	11 (44)	
IV	16 (12)	7 (7)	9 (36)	
Tumor size				
<5 cm	6 (5)	4 (4)	2 (8)	0.20
5–10 cm	36 (28)	26 (25)	10 (40)	
>10 cm	85 (67)	72 (71)	13 (52)	
Ascites				
No	51 (40)	45 (44)	6 (24)	0.07
Yes	76 (60)	57 (56)	19 (76)	
Residual tumor				
No	91 (72)	85 (83)	6 (24)	**<0.0001**
Yes	36 (28)	17 (17)	19 (76)	
CA125				
Normal	30 (24)	27 (28)	3 (12)	0.11
Elevated	93 (76)	71 (72)	22 (88)	

*P*-values with statistical significance are bolded.

* *P*-values derived from χ^2^ test; for statistical significance, FDR (false discovery rate) testing by the Benjamini-Hochberg method was applied.

The majority of the p53 normal cases were stage I (69%), whereas only a minority (16%) of the p53 abnormal cases were limited to the ovaries. No residual tumor was present in the primary surgery in 83% of p53 normal carcinomas, whereas in the p53 abnormal group, residual tumor was present in 76% of the cases. There was no significant difference in the distribution of patient age, tumor size, ascites, or CA125 level between p53 normal and p53 abnormal carcinomas (Table 1).

### Survival in p53 Normal Versus p53 Abnormal Groups

**FIGURE 1:**
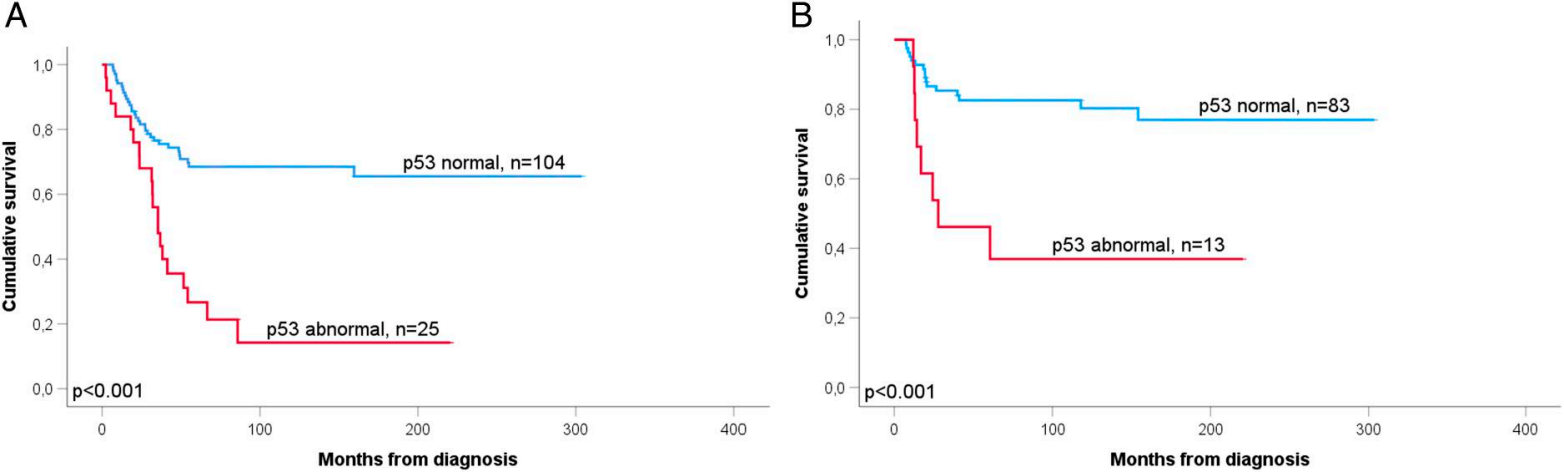
Disease-specific overall survival (A) and disease-free survival (B) in p53 normal and p53 abnormal groups.

The p53 abnormal group had significantly worse disease-specific overall survival (DSS) than the p53 normal group (*P*<0.001) (Fig. 1A). The p53 abnormal group also had significantly poorer disease-free survival (DFS) (*P*<0.001) (Fig. 1B).

**FIGURE 2:**
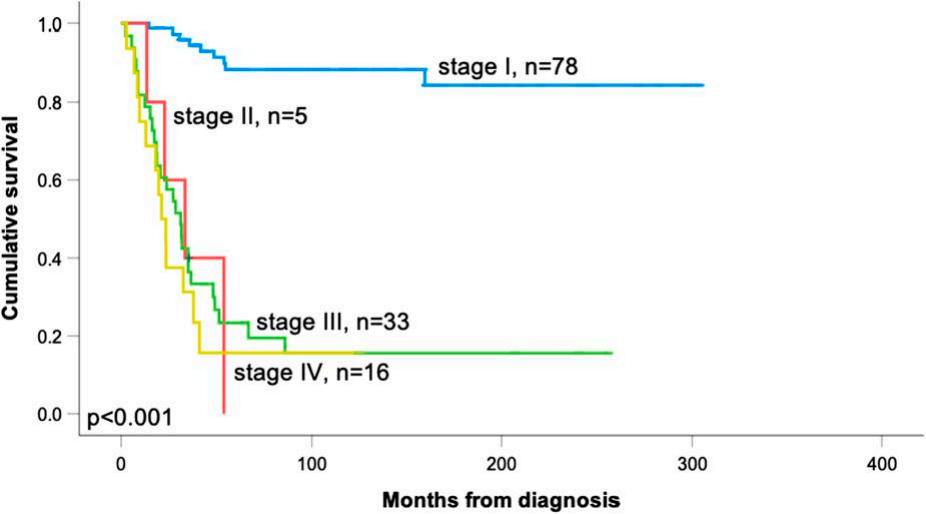
Disease-specific overall survival in different stages (both p53 normal and p53 abnormal clear cell carcinomas).

**FIGURE 3:**
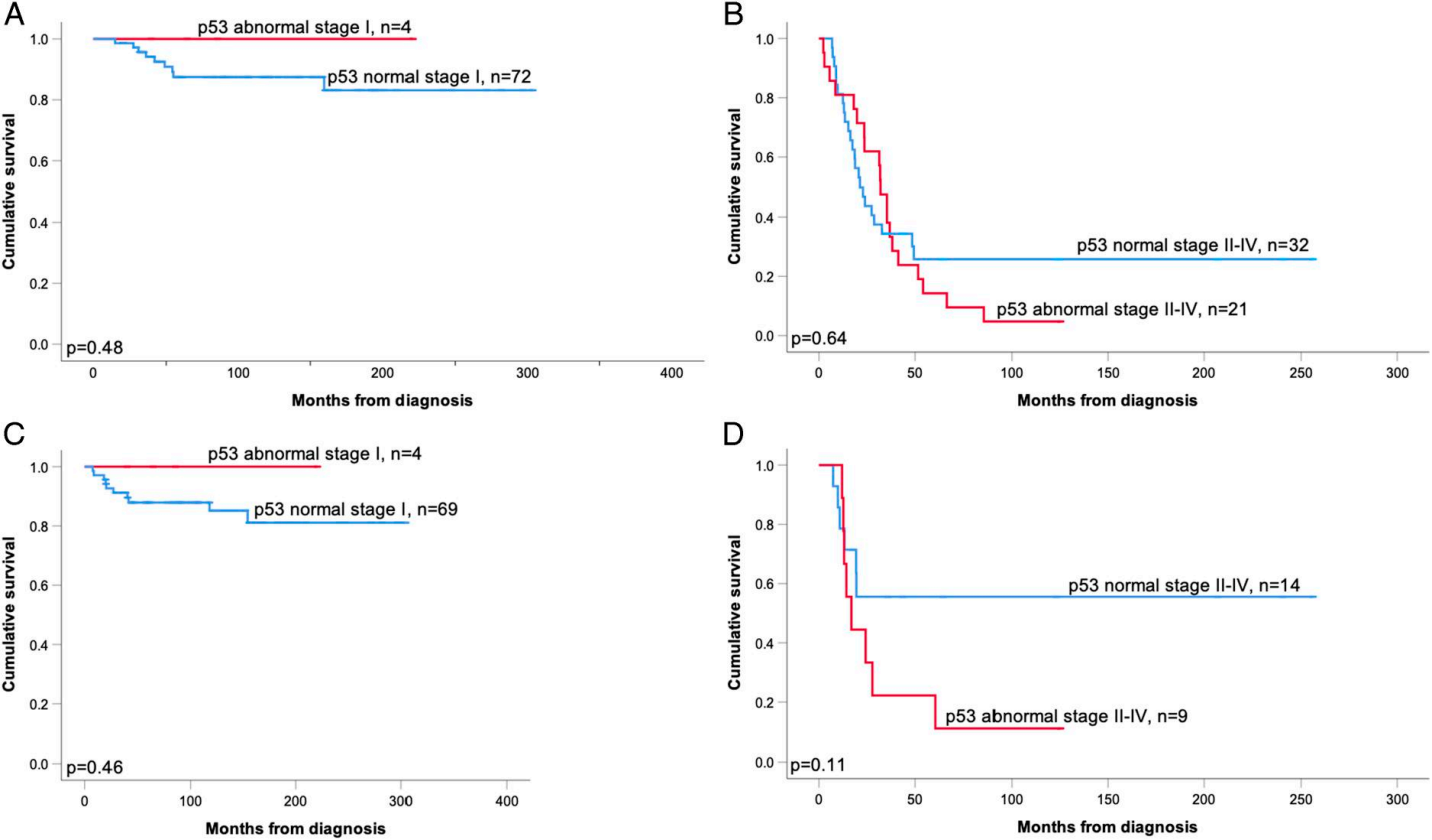
Disease-specific overall survival (A, B) and disease-free survival (C, D) in different stages and p53 groups.

The DSS in all cases in different stages (I–IV) is presented in Figure 2. The survival was clearly better in stage I as compared with the other stages (II–IV). However, there was no significant difference in survival between stages II–IV, so these were combined for further analyses as one group (St II–IV). The DSS and DFS in different stages and p53 groups can be seen in Figure 3 (A–B and C–D, respectively). We found no significant difference in DSS or DFS between the p53 normal and abnormal groups when the analyses were separated according to stage (I vs. II–IV). However, the number of p53 abnormal stage I patients was small (n=4), which may influence the comparison analysis.

### The Association of Clinicopathologic Characteristics With Survival in p53 Normal and p53 Abnormal Groups

**FIGURE 4:**
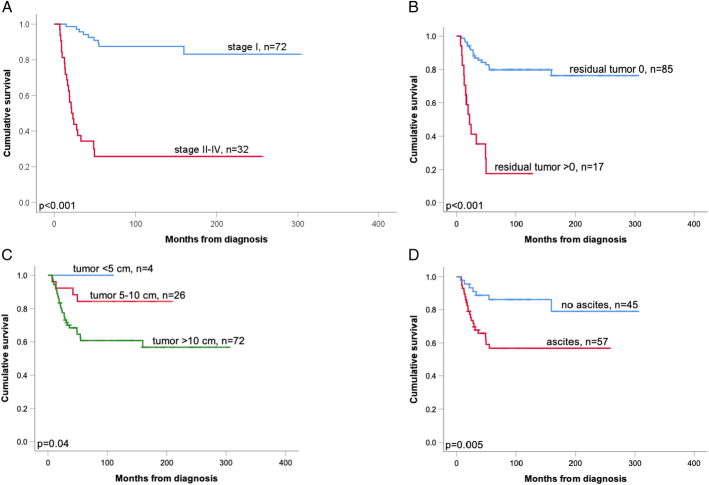
Disease-specific overall survival in p53 normal clear cell ovarian carcinoma patients according to (A) stage, (B) residual tumor, (C) tumor size, and (D) presence of ascites.

The prognostic value of clinicopathologic factors was analyzed separately for p53 normal and p53 abnormal clear cell ovarian carcinomas. In p53 normal carcinomas, higher disease stage, residual tumor at primary surgery and presence of ascites were associated with shorter DSS (Fig. 4, Table 2). Tumor size and CA125 serum value were not significantly associated with DSS, but there was a tendency for association (*P*=0.04 and 0.05, respectively; the cut-offs with Benjamini-Hochberg method *P*=0.025 and 0.029, respectively).

**TABLE 2:** Clinicopathological Characteristics and Their Associations With Disease-Specific Overall Survival (DSS) in p53 Normal and p53 Abnormal Groups

		Association With DSS (*P*)^*^	
Clinicopathologic Factor	No. Cases, n (%)	p53 Normal	p53 Abnormal
Age at diagnosis			
<58 yr (median)	64 (50)	NS	NS
≥58 yr (median)	65 (50)		
Disease stage			
I	76 (59)	***P*** **<0.001**	***P*** **=0.005**
II–IV	53 (41)		
Tumor size			
<5 cm	6 (5)	*P*=0.04	NS
5–10 cm	36 (28)		
>10 cm	85 (67)		
Ascites			
No	51 (40)	***P*** **=0.005**	NS
Yes	76 (60)		
Residual tumor			
No	91 (72)	***P*** **<0.001**	***P*** **<0.001**
Yes	36 (28)		
CA125			
Normal	30 (24)	*P*=0.05	NS
Elevated	93 (76)		

*P*-values with statistical significance are bolded.

* *P*-values derived from the log-rank test; for statistical significance, FDR (false discovery rate) testing by the Benjamini-Hochberg method was applied.

**FIGURE 5:**
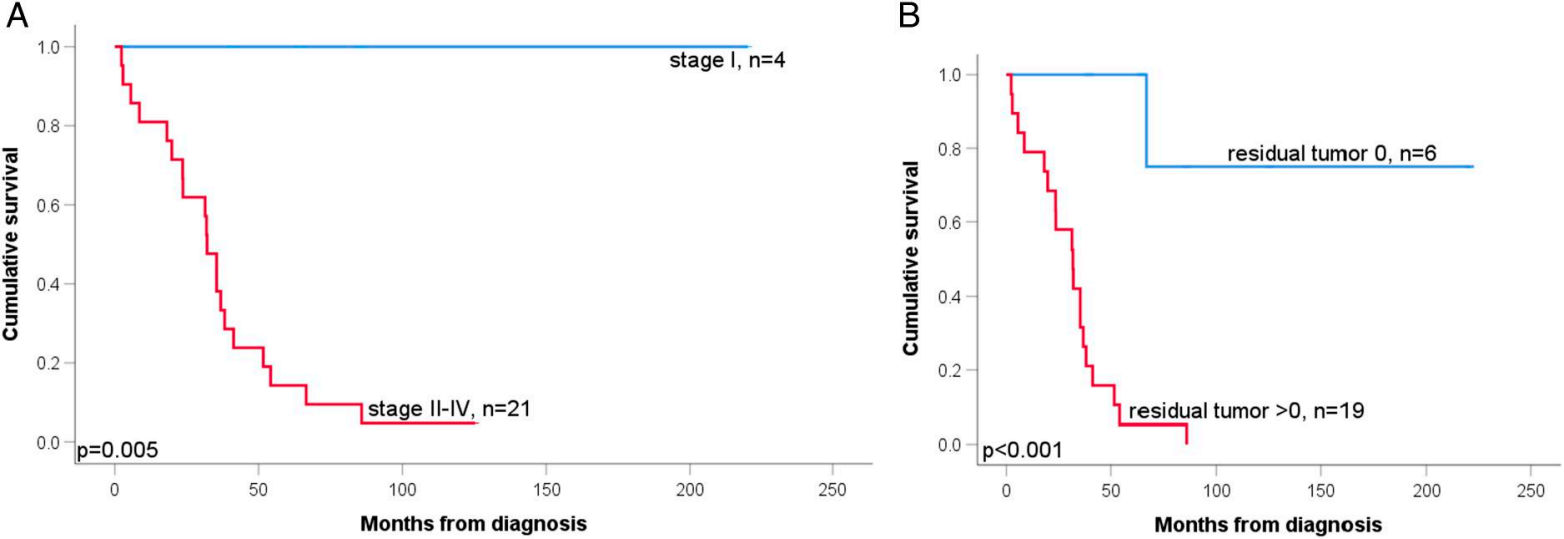
Disease-specific overall survival in p53 abnormal clear cell ovarian carcinoma patients according to (A) stage and (B) residual tumor.

For p53 abnormal tumors, similar associations were found only for stage and residual tumor (Fig. 5, Table 2). In both p53 normal and p53 abnormal tumors, a significant association with shorter disease-free survival (DFS) was found for higher disease stage and residual tumor at primary surgery (for the p53 normal group, *P*=0.003 for both and for the p53 abnormal group, *P*=0.01 and 0.009 accordingly) (Table 3).

**TABLE 3:** Clinicopathologic Characteristics and Their Association With Disease-Free Survival (DFS) in p53 Normal and p53 Abnormal Groups

		Association With DFS (*P*)^*^	
Clinicopathologic Factor	No. Cases, n (%)	p53 Normal	p53 Abnormal
Age at diagnosis			
<58 yr (median)	48 (50)	NS	NS
≥58 yr (median)	48 (50)		
Disease stage			
I	73 (76)	***P*** **=0.003**	***P*** **=0.01**
II-IV	23 (24)		
Tumor size			
<5 cm	6 (6)	NS	NS
5–10 cm	29 (31)		
>10 cm	60 (63)		
Ascites			
No	46 (48)	NS	NS
Yes	49 (52)		
Residual tumor			
No	84 (88)	***P*** **=0.003**	***P*** **=0.009**
Yes	12 (12)		
CA125			
Normal	28 (30)	NS	NS
Elevated	65 (70)		

*P*-values with statistical significance are bolded.

* *P-*values derived from the log-rank test; for statistical significance, FDR (false discovery rate) testing by the Benjamini-Hochberg method was applied.

### Expression of the Biomarkers in p53 Normal Versus p53 Abnormal Groups

The frequencies of expression of the different markers are represented in Table 4. Significant differences between p53 normal and p53 abnormal tumors were seen in the expression of ER, ARID1A, HNF1-β, and p16.

**TABLE 4:** Expression of the Biomarkers in p53 Normal and p53 Abnormal Tumors

	Total, n/N (%)	p53 Normal, n (%)	p53 Abnormal, n (%)	*P* ^*^
No. patients	129	104 (81)	25 (19)	
PR positivity	5/127 (4)	4 (4)	1 (4)	0.99
ER positivity	31/128 (24)	20 (19)	11 (44)	**0.01**
Nuclear β-catenin	0/126 (0)	0 (0)	0 (0)	—
Vimentin positivity	65/128 (51)	53 (52)	12 (48)	0.76
ARID1A loss	63/129 (49)	58 (56)	5 (20)	**0.001**
HNF1-β positivity	107/129 (83)	96 (92)	11 (44)	**0.0001**
E-cadherin loss	2/129 (2)	1 (1)	1 (4)	0.27
HER2 positivity	36/128 (28)	29 (28)	7 (28)	0.99
MIB-1 ≥30%	61/126 (48)	44 (43)	17 (71)	0.09
p16 overexpression	20/127 (16)	10 (10)	10 (40)	**0.0001**
L1CAM positivity	27/127 (21)	22 (22)	5 (20)	0.86
MMR abnormality	5/126 (4)	5 (5)	0 (0)	0.27

*P*-values with statistical significance are bolded.

* *P*-values derived from the χ^2^ test; for statistical significance, FDR (false discovery rate) testing by the Benjamini-Hochberg method was applied.

The frequency of ER positivity was 24% in the whole clear cell carcinoma cohort. Of the p53 normal tumors, 19% were ER positive, whereas the frequency was 44% in the p53 abnormal tumors (*P*=0.01). The frequency of PR positivity was low in the whole cohort (4%), and there was no difference between the subgroups.

ARID1A loss was found in 49% of all clear cell carcinomas: the percentage was 56% in the p53 normal and 20% in the p53 abnormal group (*P*=0.001).

HNF1-β positivity was found in 83% of the clear cell carcinomas. It was more common in p53 normal tumors (92%) than in p53 abnormal tumors (44%) (*P*=0.0001).

Altogether, 16% of the clear cell carcinomas presented with p16 overexpression. The percentage was higher in the p53 abnormal (40%) than in the p53 normal group (10%) (*P*=0.0001).

### Association of the Biomarkers With Survival in p53 Normal and p53 Abnormal Groups

**FIGURE 6:**
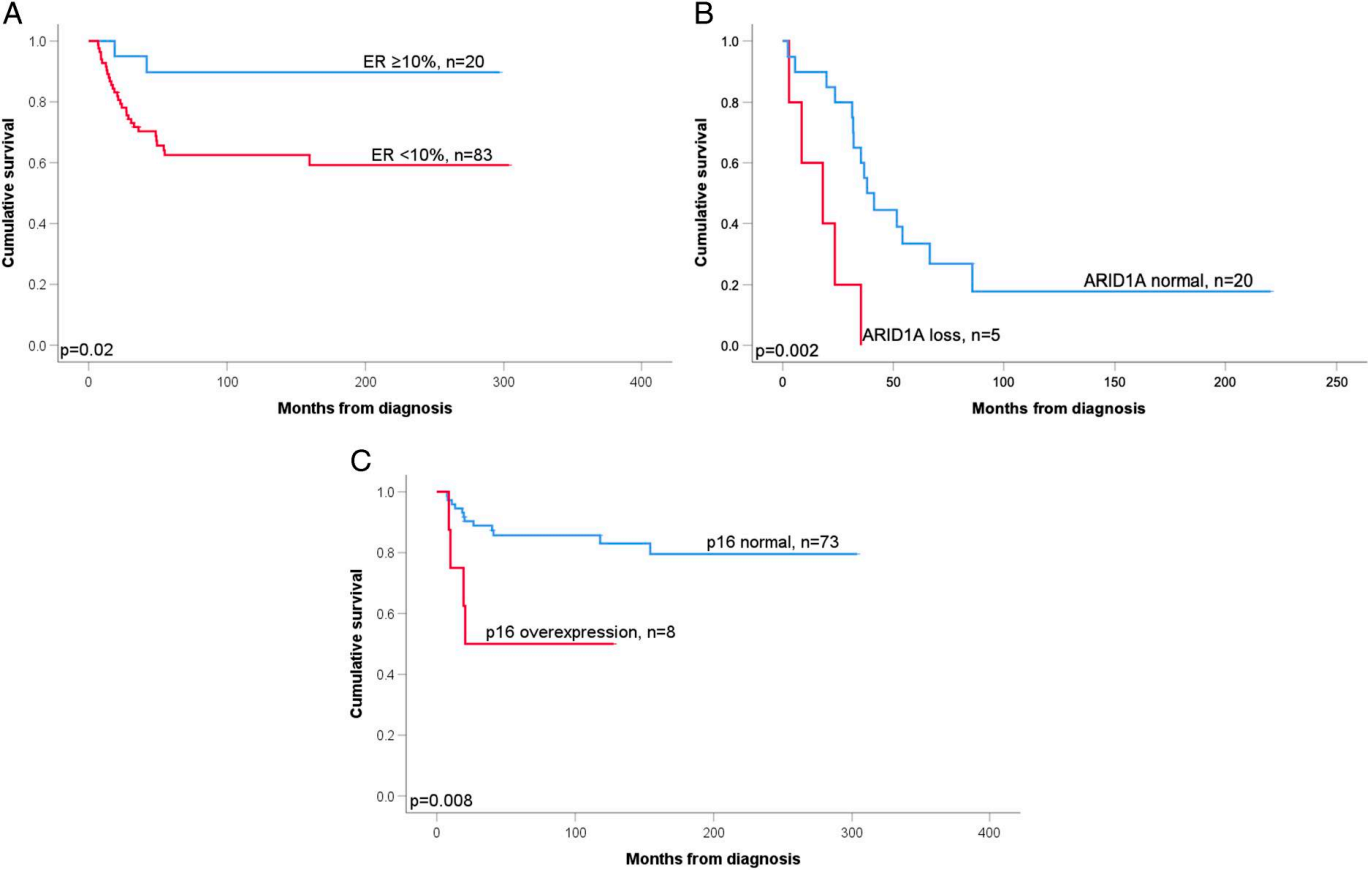
(A) Disease-specific overall survival in the p53 normal group according to ER status. (B) Disease-specific overall survival in the p53 abnormal group according to ARID1A status. (C) Disease-free survival (DFS) in the p53 normal group according to p16 status.

The significant associations of different biomarkers with DSS and DFS are depicted in Figure 6. In the p53 normal carcinomas, ER positivity was associated with longer DSS (*P*=0.02) (Fig. 6A) and p16 overexpression with shorter DFS (*P*=0.008) (Fig. 6C). Interestingly, in the p53 abnormal group, ARID1A loss was associated with worse DSS (*P*=0.002) (Fig. 6B).

### Multivariate Survival Analysis in p53 Normal and p53 Abnormal Groups

In multivariate analysis for DSS, only stage remained an independent prognostic factor in both the p53 normal (*P*<0.001, 95% CI, 1.86–3.46) and p53 abnormal (*P*=0.008, 95% CI, 1.22–3.63) groups. In multivariate analysis of DFS, stage had independent prognostic value in the p53 normal group (*P*=0.01, 95% CI, 1.13–2.79). None of the other clinicopathologic or biomarkers presented with independent prognostic significance.

### Association With Response to Primary Therapy

p53 abnormality was significantly associated with incomplete response to primary treatment (surgery and adjuvant chemotherapy) (*P*<0.003). Complete response was found in 81% of the cases in the p53 normal group as compared with 52% in the p53 abnormal group. This difference was largely explained by the majority of the p53 normal tumors being of early stage (73%) and p53 abnormal tumors being of late stage (27%).

In addition to lowerstage, clinical factors that associated with complete response after primary therapy in the p53 normal group were smaller tumor size and the absence of residual tumor (*P*=0.04 and <0.001, respectively). Elevated CA125 and presence of ascites were associated with incomplete response to therapy (*P*=0.03 and 0.02, respectively). In the p53 abnormal group, lower stage and the presence of no residual tumor were associated with complete response after primary therapy (*P*=0.02 and 0.007, respectively). No significant associations were found between biomarkers and response to therapy in the p53 normal or p53 abnormal groups.

To evaluate the actual response to adjuvant chemotherapy, response to primary therapy was also analyzed separately in the cases that had residual tumor in primary surgery. No significant difference was found between the p53 normal and p53 abnormal groups (*P*=0.64): 29% of the p53 normal tumors and 37% of the p53 abnormal tumors had a complete response after primary therapy.

### Association of Biomarkers With Clinicopathologic Factors

Associations between different biomarkers and clinicopathologic factors were also investigated in p53 normal and p53 abnormal groups. In the p53 normal group, a significant association was found between L1CAM expression and residual tumor in primary surgery. The patients with residual tumor in primary surgery were more likely to have L1CAM-expressing tumors than patients without residual tumor (41% and 18%, respectively) (*P*=0.04).

No significant associations were found between other biomarkers and clinicopathologic factors.

## DISCUSSION

Clear cell ovarian carcinoma accounts for 5% to 10% of ovarian carcinomas and, when diagnosed at an advanced stage, confers a poor prognosis. Unlike high-grade serous carcinoma, whose treatment has progressed significantly via HRD testing and availability of PARP inhibitors, the molecular subtyping and targeted treatments have been lacking in CCOC. Bolton et al.^2^ proposed a model comprising 2 subclasses of CCOC: the first group (83%) characterized by canonical CCOC gene alterations (eg, *ARID1A* mutations), and the second consisting predominantly of *TP53*-mutated tumors (17%). In addition, independent prognostic value for p53 expression has been reported by the international consortium analysis of CCOC.^4^ We evaluated the clinical correlations of the proposed new subclassification in a well-characterized cohort of 129 CCOCs treated at a single institution. Indeed, our results confirmed the earlier sequencing-based findings at the immunohistochemical level: tumors with abnormal p53 staining (19%) demonstrated markedly worse 5-yr disease-specific overall survival and disease-free survival compared with the p53-normal group (81%). In analyses of clinical factors, the p53-abnormal group was associated with advanced stage at diagnosis and the presence of residual tumor following primary surgery. No correlation was observed with age, tumor size, presence of ascites, or CA125 serum level. When the analyses were separated according to stage, no significant difference in DSS or DFS was seen between p53 normal and p53 abnormal groups (stage I and stage II–IV separately). Thus, the poorer outcomes observed in the p53-abnormal group appear to be largely attributable to their higher stage at presentation and the more frequent presence of macroscopic residual disease.

The p53 normal and p53 abnormal subgroups were compared with respect to the expression of 12 selected biomarkers: the p53 normal cases presented with higher frequency of ARID1A loss (56% vs. 20%, *P*=0.001) and HNF1-β positivity (92% vs. 44%, *P*=0.001), whereas p16 overexpression (40% vs. 10%, *P*=0.001) and ER positivity (44% vs. 19%, *P*=0.01) were more common in p53 abnormal cases. The observation of ARID1A loss is consistent with the findings of Bolton and colleagues, in which the *TP53* wild-type group was enriched for *ARID1A* mutations and other canonical CCOC gene alterations such as *PIK3CA* or *TERT* mutations. Genome-wide DNA methylation-based analysis of 271 CCOCs also defined 2 clusters, of which cluster 1 showed association with *TP53* mutation/aberrant p53 expression and cluster 2 aneuploidy and *ARID1A/PIK3CA* mutations. Cluster 1 cases had more advanced disease, macroscopic residual tumor and poorer survival as compared with cluster 2, similar to our findings.^13^ The existence of these 2 distinct clusters has been verified by another genome-wide methylation study of CCOC, which demonstrated a set of genes differentially methylated in the *ARID1A*-mutated type rather than showing global DNA methylation changes.^14^

To further elucidate the behavior of these 2 subgroups of CCOC, we evaluated the prognostic value of clinical factors separately in p53 normal and aberrant groups. In both groups, the most significant correlations were associations of stage and residual tumor with poor prognosis. This underlines the significance of no residual disease at primary surgery in clear cell histotype, which was recently found to benefit the most from complete surgical resection.^15^ Interestingly, we found that the presence of ascites was associated with shorter DSS in p53 normal, but not in p53 abnormal cases. In addition, there was a tendency toward poorer survival with increasing tumor size in the p53 normal cases. The association of tumor size with prognosis has also been reported by others in molecularly unselected stage I cases,^16,17^ but, to our knowledge, not across all stages of CCOC.

Clear cell carcinoma is typically recognized for its intrinsic resistance to chemotherapy.^18^ To evaluate potential differences in treatment response between the 2 subgroups, we examined outcomes of primary therapy among cases with residual tumor after primary surgery. No significant differences were observed between the groups: complete response rates to primary therapy were poor in both the p53 normal and p53 abnormal tumors (29% and 37%, respectively). This aligns with the previous findings in clear cell carcinoma overall.^18^ In the p53 abnormal group, the response rate was significantly lower than in high-grade serous carcinoma (typically 70%–80%), thus reinforcing the evaluation of histology in these cases, being true CCOC, rather than misclassified HGSCs.

Given the biological differences between the p53-related subgroups, we analyzed the prognostic value of 12 immunohistochemical markers separately within the 2 subclasses. Interestingly, ARID1A loss was associated with poor overall survival in the p53 abnormal group, but none of the other markers were associated with outcome in this group. In the p53 normal group, ER positivity correlated with improved overall survival and p16 positivity with worse disease-free survival. PR positivity was low in both subclasses (4%) and showed no relationship with survival. As previously stated, ARID1A mutation/loss has been generally linked to improved outcome.^2,13^ However, a meta-analysis of 12 IHC studies has shown contradicting results, indicating that reduced ARID1A expression is associated with diminished overall survival.^19^ Overexpression of p16 has been linked to poor prognosis in CCOC,^20,21^ whereas, to our knowledge, no prognostic significance of ER expression in CCOC has been reported previously. The previous studies have analyzed CCOC as one entity, which limits direct comparison with our results. Dividing histotypes into subgroups may enable finding new, unexpected biologically relevant correlations.

L1CAM was the only marker to present associations with other clinical factors other than survival. In our previous work^6^ L1CAM expression was associated with larger tumor size in clear cell ovarian carcinomas. This association was not observed in the current study when the tumors were separated into the 2 p53-defined subgroups. However, we found a significant association of L1CAM expression with residual tumor in the p53 normal group. To our knowledge, no other studies on L1CAM in CCOC have been published. In endometrial carcinoma, L1CAM is associated with poor prognostic variables and outcome, especially in the endometrioid p53wt/NSMP group.^22,23^

We have previously analyzed the same set of immunohistochemical biomarkers in another endometriosis-associated ovarian cancer, endometrioid carcinoma.^24^ As expected, the prevalence of hormone receptor positivity was higher in endometrioid carcinoma than in clear cell carcinoma (PR 68% vs. 4% and ER 85% vs. 24%), whereas HNF1-β positivity was more common in clear cell tumors than in endometrioid tumors (83% vs. 42%). A recent study on endometriosis-associated cancers has attributed these differences between histologic types to their “cell state of origin”: although clear cell and endometrioid carcinomas likely arise from the same cell type, they appear to activate distinct differentiation programs—secretory in clear cell carcinoma and proliferative in endometrioid carcinoma.^25^ In our previous work on endometrioid ovarian carcinomas, nuclear β-catenin expression was associated with good prognosis and was observed in 27% of the cases. In contrast, nuclear β-catenin expression was not detected in any of the clear cell carcinomas. *CTNNB1* mutations are known to be typical of endometrioid ovarian carcinomas, and our finding underlines the difference between the histologic types also at the IHC level. However, HER2 positivity was more common in clear cell than in endometrioid carcinomas (28% vs. 4%), suggesting a potential additional treatment option for patients with clear cell carcinoma.

A limitation of this study is the relatively small number of p53 abnormal cases (n=25), which decreases the statistical power of the analyses. Consequently, results reported as nonsignificant may not necessarily reflect a true absence of association. Larger studies are therefore needed to further explore and validate these findings. The DFS analysis should also be regarded as a secondary endpoint, whereas DSS represents the primary survival endpoint. DFS includes only those patients who achieved a complete response following primary treatment (surgery and chemotherapy), and thus reflects recurrence rates within this more favorable subgroup.

To conclude, in endometrial carcinoma, the TCGA-based classification divides the disease into 4 pathogenetically and clinically relevant subtypes. In CCOC, POLE mutation and MMRd are rare, suggesting that the classification is different. Our findings confirm the existence of 2 separate molecular subgroups of clear cell ovarian carcinomas, p53 normal and p53 abnormal, with different characteristics and patient prognosis. Clinical and molecular differences were identified within these subgroups. The analyses were based on immunohistochemistry, a method readily applicable in routine clinical practice. To design clinical trials testing new therapies, it is essential to assess different subgroups as distinct entities to identify the most effective treatments for each group. Currently, several studies are evaluating immunotherapy in clear cell ovarian cancer. However, the reported relationships between *ARID1A* or *TP53* mutations and immunotherapy response have been inconsistent, underscoring the need for further investigation. Additional research is required to identify new potential therapeutic targets for this challenging histotype.

## Supplementary Material
