# Supplementary material for: p53 Normal and p53 Abnormal Clear Cell Ovarian Carcinomas: Clinical Characteristics and Biomarker Profiles
Source: Int J Gynecol Pathol. 2026 May 27;45(5):340–8. doi: 10.1097/PGP.0000000000001191 (PMC13460317; doi:10.1097/PGP.0000000000001191)

Supplemental Digital Content 2. Examples of immunohistochemical stainings for different biomarkers.

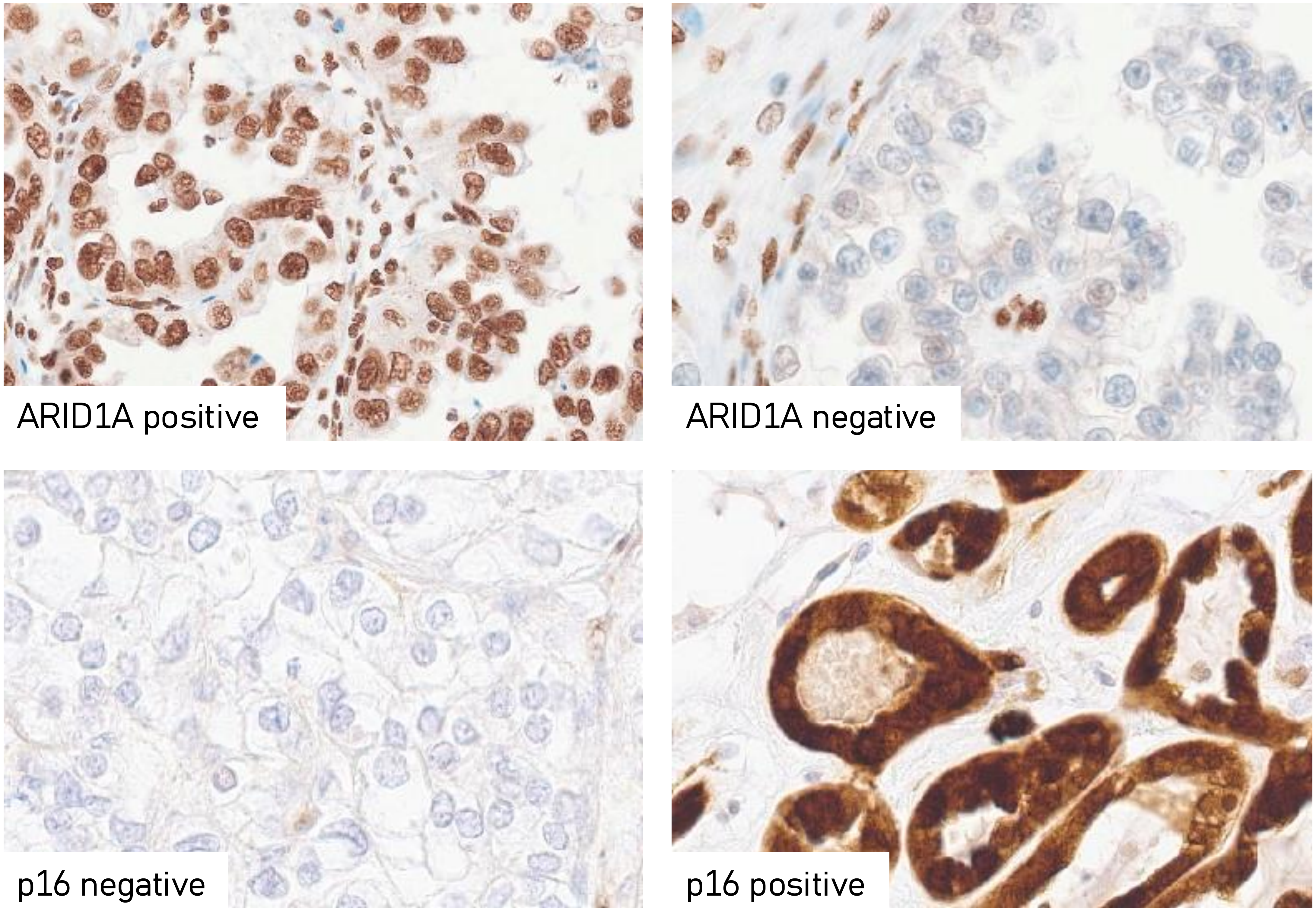

Supplement: Supplementary file 2 [file pgp-45-340-s002.pdf]
